# The Bacterial Amyloid-Like Hfq Promotes *In Vitro* DNA Alignment

**DOI:** 10.3390/microorganisms7120639

**Published:** 2019-12-03

**Authors:** Frank Wien, Denis Martinez, Etienne Le Brun, Nykola C. Jones, Søren Vrønning Hoffmann, Jehan Waeytens, Melanie Berbon, Birgit Habenstein, Véronique Arluison

**Affiliations:** 1 Synchrotron SOLEIL, 91192 Gif-sur-Yvette, France; 2 Institute of Chemistry and Biology of Membranes and Nano-objects, CBMN UMR5248 CNRS Université de Bordeaux INP, 33607 Pessac, France; d.martinez@iecb.u-bordeaux.fr (D.M.); m.berbon@iecb.u-bordeaux.fr (M.B.); b.habenstein@iecb.u-bordeaux.fr (B.H.); 3 Laboratoire Léon Brillouin LLB, CEA, CNRS UMR12, Université Paris Saclay, CEA Saclay, 91191 Gif-sur-Yvette, France; etiennetheo.lebrun@gmail.com; 4 ISA, Department of Physics and Astronomy, Aarhus University, 8000 Aarhus C, Denmark; nykj@phys.au.dk (N.C.J.); vronning@phys.au.dk (S.V.H.); 5 Structure et Fonction des Membranes Biologiques, Université libre de Bruxelles, B1050 Bruxelles, Belgique; Jehan.Waeytens@ulb.be; 6 Laboratoire de Chimie Physique d’Orsay, CNRS UMR8000, Université Paris-Sud, Université Paris-Saclay 91400 Orsay, France; 7 Université de Paris, UFR Sciences du vivant, 35 rue Hélène Brion, 75205 Paris cedex, France

**Keywords:** Nucleoid-associated protein (NAP), DNA compaction, DNA recombination, amyloid, Sm protein, Synchrotron Radiation Circular and Linear Dichroism (SRCD/SRLD), Couette flow cell, atomic force microscopy (AFM), solid state NMR (ssNMR)

## Abstract

The Hfq protein is reported to be involved in environmental adaptation and virulence of several bacteria. In Gram-negative bacteria, Hfq mediates the interaction between regulatory noncoding RNAs and their target mRNAs. Besides these RNA-related functions, Hfq is also associated with DNA and is a part of the bacterial chromatin. Its precise role in DNA structuration is, however, unclear and whether Hfq plays a direct role in DNA-related processes such as replication or recombination is controversial. In previous works, we showed that *Escherichia coli* Hfq, or more precisely its amyloid-like C-terminal region (CTR), induces DNA compaction into a condensed form. In this paper, we evidence a new property for Hfq; precisely we show that its CTR influences double helix structure and base tilting, resulting in a strong local alignment of nucleoprotein Hfq:DNA fibers. The significance of this alignment is discussed in terms of chromatin structuration and possible functional consequences on evolutionary processes and adaptation to environment.

## 1. Introduction

Hfq is a highly abundant protein present in half of bacterial species [1]. Originally identified for its involvement in bacteriophage Qβ replication (which inspired its name, host factor for bacteriophage Qβ [2]), Hfq is now recognized as an RNA chaperone that interacts with many RNAs and plays crucial roles in riboregulation. Indeed, the protein coordinates multiple roles inside bacteria, consistent with its high level of expression (cellular concentration about 60 µM) [3]. The pleiotropic function of Hfq was determined when its gene (*hfq*) was disrupted in *Escherichia coli*, yielding various phenotypes. These include changes in growth and mutagenesis rates, stress resistance, or plasmid supercoiling [4,5]. Most of these *hfq*-null phenotypes are attributed to the role of Hfq in small noncoding RNA (sRNA) based regulation [6,7]. This regulatory mechanism is based on the annealing of the sRNA to a target mRNA [8]. Mostly, the sRNA hybridization occurs around the ribosome binding site (rbs) and/or AUG start codon and prevents the initiation of translation, therefore altering both mRNA translation and stability [6,9,10]. Importantly, a significant part of *hfq*-null phenotypes are due to the role of Hfq in the expression of stress sigma factor σS-dependent genes [11].

In addition to its role in post-transcriptional regulation, Hfq has also been described as being capable of binding DNA and identified as one of the *E. coli* nucleoid associated proteins (NAP), that have an essential role in the organization of bacterial chromatin [12,13,14,15]. This was notably revealed by cellular localization experiments demonstrating that about 20% of Hfq was complexed with DNA in the nucleoid, corresponding to an average local concentration of ≈10–15 µM [15,16,17]. In the nucleoid, Hfq appears to have a heterogeneous localization as opposed to the more uniform distribution of most nucleoid proteins such as H-NS (histone-like nucleoid structuring protein), HU (heat-unstable nucleoid protein), IHF (integration host factor), StpA (suppressor of td-phenotype A), and Dps (DNA-binding protein from starved cells) [16]. Thus, Hfq concentration may locally increase from tens of µM to hundreds of µM.

Even though few studies have shed light on the role of Hfq in DNA metabolism, its direct or indirect role in DNA related processes is now firmly established [15]. Hfq has been shown to influence DNA supercoiling and compaction [4,18,19]. Some studies have also suggested a role for the protein in replication, transcription, and transposition efficiency [20,21,22,23,24]. Some of the phenotypic effects due to *hfq* mutation may indeed be due to defects in DNA-related processes; for instance, slow-down of growth and filamentation are signs of replication misregulation. Sensitivity to mutagens could also indicate problems in DNA repair [4,25].

Structurally, Hfq is related to the Sm eukaryotic family of proteins, which participate in RNA-related processes such as splicing [26,27]. Indeed, the amino-terminal region of Hfq (NTR, ≈65 amino acid residues) is comprised of an antiparallel bent β-sheet capped by an N-terminal α-helix and folds similarly to Sm proteins. In addition, also similarly to Sm proteins, the β-sheets of monomers interact with each other to assemble in a toroidal structure (hexameric for Hfq) [6,28]. Besides its Sm-like NTR domain, Hfq also presents a C-terminal region (CTR). Our previous results highlighted the protein propensity to compact DNA mainly due to its CTR [18,19,29]. The atomic structure of Hfq CTR remains unknown, because all 3D-structures resolved for various Hfq lack this region [30,31,32,33,34]. In the structure of Hfq bound to RydC sRNA, solved by X-ray crystallography, the full-length protein was used but only the Sm part was observed and the CTR could not be resolved (PDB ID 4V2S) [35]. Indeed, Hfq CTR self-assembles and forms amyloid-like structures *in vitro* [18,36,37]. Furthermore, Hfq self-assembly is also observed *in vivo* [38,39,40]. An obstacle towards obtaining a 3D structure of the Hfq CTR could thus be due to its propensity for forming amyloid-like assemblies [41]. Nevertheless, this self-assembly is functionally important as it is at the origin of Hfq-mediated DNA bridging and the resulting compaction [18,29].

The work reported here further explores how the Hfq CTR self-assembles on DNA, resulting in mesoscopically well-ordered structures, which induce a strong local alignment of DNA. We show that the local organization of the DNA strands induced by the Hfq-CTR offers a very efficient surface area exposition of both DNA and Hfq-CTR, leading to an optimal molecular association. Examination of Hfq-CTR fibrillar ultrastructures by Synchrotron Radiation Circular and Linear Dichroism (SRCD/SRLD) spectroscopies, atomic force microscopy (AFM), and solid-state NMR (ssNMR) enables us to propose a new mode for DNA structuring in the nucleoid and is presented herein.

## 2. Materials and Methods

### 2.1. Preparation of the Complexes for SRCD, SRLD, and AFM

The peptide corresponding to the CTR region of Hfq (residues 64–102, referred to as Hfq-CTR throughout the manuscript) was synthetized by Proteogenix SA (Schitilgheim, France). The sequence of the peptide is SRPVSHHSNNAGGGTSSNYHHGSSAQNTSAQQDSEETE (the amyloid region is underlined, [42]). Single strand dA_59_ and dT_59_ oligonucleotides solutions (Eurogentec, Seraing, Belgium) were mixed together and heated at 90 °C for 2 min to form the (dA:dT)_59_ duplex. This model was chosen because of the significant affinity of Hfq for AT-rich sequences and to avoid natural alignment of long DNA strands. Complexes between Hfq-CTR and the (dA:dT)_59_ duplex were prepared at 7.3 mM in water to allow optimal CD analyses in the far UV. Unless stated otherwise, the stoichiometry was 1 Hfq-CTR per one base pair. Samples were analyzed at specific times, usually 2 weeks after preparation to allow for peptide self-assembly that is not instantaneous [18].

### 2.2. Synchrotron Radiation Circular Dichroism (SRCD)

For SRCD analysis, measurements and data collection were carried out on the DISCO beam-line at SOLEIL Synchrotron, France (proposals 20171061 and 20181037) [43]. First, 2–4 µL of samples were loaded into circular demountable CaF_2_ cells of 12 micron path length [44]. Three separate data collections with fresh sample preparations were carried out to ensure consistency and repeatability. Spectral acquisitions of 1 nm steps at 1.2 s integration time, between 320 and 170 nm were performed in triplicate for the samples as well as for the baselines. (+)-camphor-10-sulfonic acid (CSA) was used to calibrate amplitudes and wavelength positions of the SRCD experiment. Data-analyses including averaging, baseline subtraction, smoothing, and scaling were carried out with CDtool [45].

### 2.3. Synchrotron Radiation Linear Dichroism (SRLD)

SRLD measurements were performed at the AU-CD beam line on the ASTRID2 synchrotron, Aarhus University, Denmark (proposal ISA-19-109). The alignment of the DNA was achieved using an outer-cylinder-rotation microvolume Couette flow cell with a path length of 0.5 mm [46]. The rotation speed was 3100 rpm, with 60 µL of sample loaded into a quartz Suprasil capillary. All spectra were recorded between 180 and 350 nm in 1 nm increments, with a dwell time of 2 s per point. At least three data accumulations were made and averaged for each condition. Baselines were collected with zero rotation and subtracted from flow spectra.

### 2.4. Atomic Force Microscopy (AFM)

AFM measurements were performed on a nanoIR2S from Anasys Instrument (Goleta, CA, USA) in contact mode with silicon tips from µmasch with a spring constant around 0.03 N/m (reference HQ:CSC38/Al-BS). First, 0.5 µL of Hfq-CTR/(dA:dT)_59_ complex was deposited on a gold substrate (Sigma-Aldrich Gold coated silicon wafer 643262) and dried with nitrogen. The deposit was washed two times with 2 µL of milliQ water to remove unattached material and finally dried again with nitrogen. The data was treated with Mountains Map 7.4, images were flattened line by line and the direction obtained is based on ISO 25178.

### 2.5. Sample Preparation for Solid-State NMR Spectroscopy

To perform NMR spectroscopy, the concentration of the (dA:dT)_59_ dsDNA sample was adjusted to 1 mM with H_2_O/D_2_O (90/10) and DSS was added for chemical shift referencing. Then, 30 mg of Hfq-CTR peptide was dissolved in pure milliQ water to reach a concentration of 50 mg/mL and vortexed vigorously until the solution became homogenous and clear. Protein filaments were assembled under agitation at room temperature for 2 weeks. For DNA-containing samples, double strand DNA was added to a solution of 30 mg Hfq-CTR in water in a dsDNA equimolar ratio and allowed to self-assemble for 2 weeks under agitation at room temperature. The stoichiometry was one Hfq-CTR per one base pair. All the samples were pelleted by centrifugation (1 h, 20,000 × *g*) and inserted into a solid-state NMR rotor (3.2 mm rotor diameter) before data acquisition.

### 2.6. Solid-State NMR Spectroscopy

ssNMR experiments were performed on a 600 MHz (14.1 T) Bruker Avance III NMR spectrometer (Bruker Biospin, Rheinstetten, Germany) using a 3.2 mm MAS probe. The MAS frequency was set to 11 kHz and the sample temperature was adjusted to 10 °C according to the DSS signals as internal reference [47]. 1D ^13^C insensitive nuclei enhanced by polarization transfer (INEPT) ssNMR experiments were recorded, detecting mobile molecule moieties. Then, 15 k scans were acquired per experiment with an acquisition time of 20 ms and an inter-scan delay of 2 s. Proton decoupling was applied during the acquisition time using the SPINAL-64 decoupling sequence [48]. Spectra were processed and analyzed using the TOPSPIN 4.0.7 software (Bruker Biospin, Rheinstetten, Germany).

### 2.7. Liquid-State NMR Spectroscopy

Solution NMR experiments were performed on an 800 MHz (18.8 T) Bruker Avance NEO NMR spectrometer equipped with a 5 mm TCI cryoprobe. 1D ^13^C solution NMR experiments were recorded at 298 K with 2 k scans per experiment with an inter-scan delay of 2 s. Spectra were processed and analyzed using the TOPSPIN 4.0.7 software (Bruker Biospin, Rheinstetten, Germany).

## 3. Results

### 3.1. DNA Structural Changes Induced by Hfq-CTR

DNA bridging by the Hfq-CTR amyloid-like region has been previously demonstrated using molecular imaging, a property that results in a strong DNA compaction *in vitro* [14,19,29]. To obtain deeper insights on the DNA:Hfq-CTR interaction we analyzed the complex using chiro-optical spectroscopy methods.

**Figure 1:**
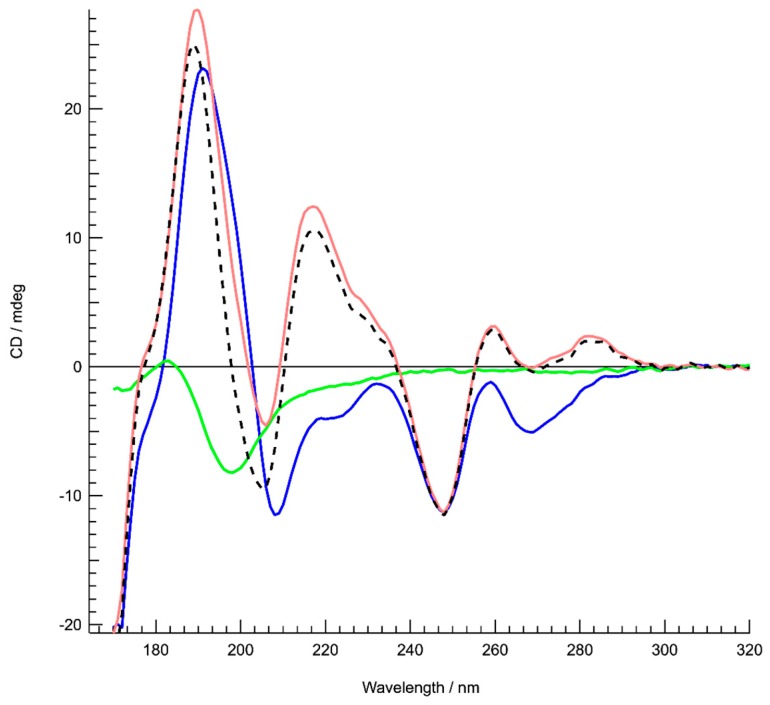
Structure characterization of DNA complexed to the Hfq-C-terminal region (CTR) by Synchrotron Radiation Circular Dichroism (SRCD) spectroscopy. Spectra of DNA in the absence (red) and presence of Hfq-CTR (blue). Hfq-CTR alone (green). The DNA spectrum (red) is identical compared to a poly[d(A)]-poly[d(T)] spectrum [49]. The spectrum of the complex (blue) is significantly different as opposed to the sum of the DNA and peptide spectra (dotted black), signifying an important conformational change of complexed DNA. In particular, inversions are observed around 220 and 285 nm. Note that the same analysis was also performed with the full-length protein. In this case we observed that Hfq has a strong propensity to align, which makes the CD analysis difficult. However, we can confirm base tilting with the inversion between 220 and 240 nm. The change in the region 260–280 nm is similar but more complex to interpret due to full length Hfq DNA melting (as shown previously in [14]) or to other complex properties of the full-length Hfq assembly.

SRCD was first used to probe how Hfq-CTR affects DNA upon binding. We identified significant spectral changes in two regions, up to 240 nm and between 250 and 300 nm (Figure 1). Assuming that the protein is restructuring upon the DNA binding [18] we identified that the DNA also changes. We identified a spectral inversion of the 220 nm CD peak, which could be correlated to the base-tilting of AT rich sequences observed before [49]. This was confirmed by comparison with spectral recordings from the literature [50].

### 3.2. Hfq-CTR Causes DNA Alignment

**Figure 2:**
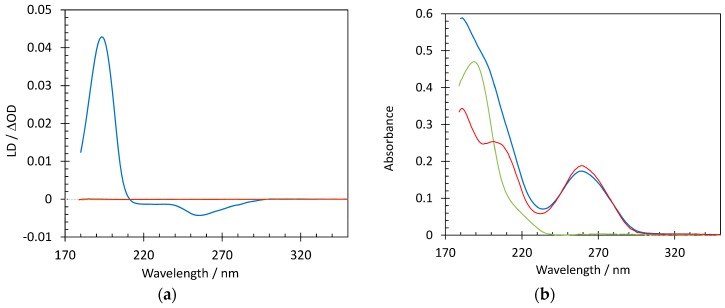
Structure characterization of DNA complexed to Hfq-CTR by Synchrotron Radiation Linear Dichroism (SRLD) spectroscopy. Spectra of DNA (0.26 mM bp) in the absence (red) and presence of Hfq-CTR (blue) and Hfq-CTR alone (green); (**a**) shows SRLD measurements, while (**b**) is the absorbance. Notice that the linear dichroism (LD) signal is essentially zero for the DNA and Hfq-CTR when not forming a complex and that similarly decreasing the peptide/DNA ratio does not result in any alignment.

Suspecting that the interaction between the Hfq-CTR and AT-rich DNA causes molecular alignment, we then turned to flow SRLD, which is a bulk method to probe polarized absorption of aligned chromophores [51]. Ultra-violet linear dichroism (LD) coupled to a microvolume Couette cell, which induces alignment of long molecules through the flow of a solution in the gap between two concentric cylinders, provides information on the orientation of chromophores of an orientable molecule such as DNA. Long DNA molecules, as e.g., observed for calf-thymus DNA [52], aligned with the flow in a Couette cell result in negative LD where the bases absorb with significant signals near 260 and 180 nm, as the base pairs are arranged perpendicular to the flow orientation. SRLD measurements show that neither Hfq-CTR alone nor the short DNA used in this work (dA:dT)_59_, result in any discernable LD signal, and hence do not align in the flow (Figure 2). However, SRLD data for (dA:dT)_59_ DNA mixed with Hfq-CTR, where all DNA is bound by protein, shows a significant signal from alignment of the complex. A negative SRLD signal arises from the DNA bases around 260 nm in the presence of Hfq-CTR, whereas the large SRLD signal near 195 nm is positive. As the SRLD from long DNA is negative, the positive signal must originate from Hfq-CTR. Proteins with β-strand secondary structure elements have a transition dipole moment near 195 nm aligned perpendicular to the direction of the strand [51]. As the SRLD signal is positive when transition moments are parallel to the alignment (flow) direction, this means that the direction of the protein secondary structure is perpendicular to the flow direction, and hence perpendicular to the DNA helix.

**Figure 3:**
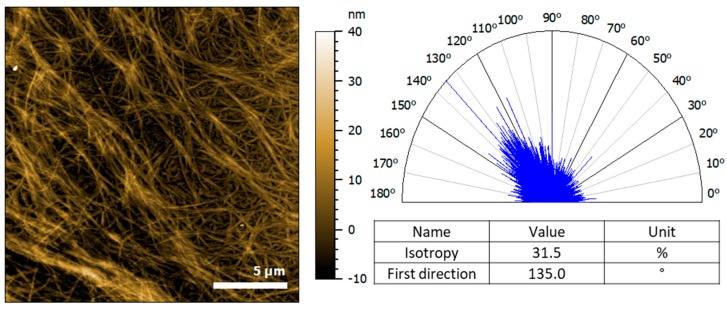
Atomic force microscopy (AFM) analysis of (dA:dT)_59_ DNA complexed to Hfq-CTR. **Left**, the height image of the fibrils deposited on the gold substrate obtained in contact mode at force constant around 30 nN. **Right**, the representation of the orientation of the fibrils analyzed with a clear preferred orientation around 135°, in contrast to Hfq-CTR alone (see Supplementary Material Figure S1).

Alignment of the Hfq-CTR: DNA complex was also confirmed by molecular imaging. AFM is a microscopic method widely used for the study of amyloid or amyloid-like assemblies, capable of detecting filamentous assemblies and their orientation at the nanoscale [53]. In the complex Hfq-CTR/(dA:dT)_59_, we clearly observe a molecular alignment of the fibrils. Figure 3 shows the height image of an assembly of complex DNA/Hfq-CTR fibrils and their orientation. Isolated fibrils have an average diameter of 10 nm, while the assemblies are composed of many fibrils wrapped together to form larger bundles. The bundle direction can be seen from the top-left corner going down to the bottom-right corner, indicating a preferred orientation around 135°. The isotropy of 31.5% shows a pronounced isotropy of the fibrils (an anisotropic surface will have a 100%). The AFM thus confirms fiber orientations detected by LD, even in the absence of flow.

### 3.3. DNA Arrangement in Hfq-CTR Filaments

**Figure 4:**
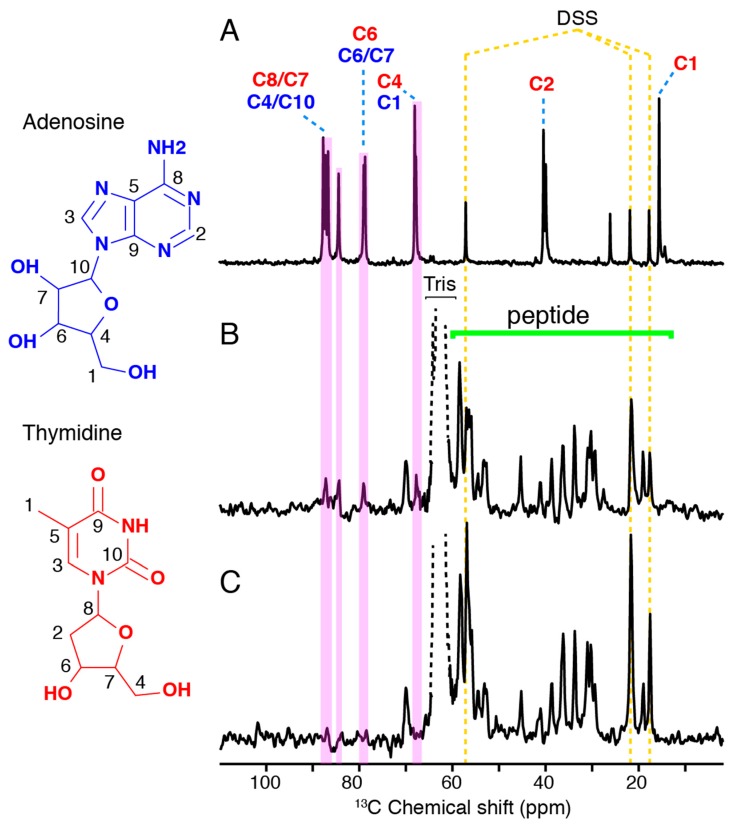
DNA association to Hfq-CTR investigated by NMR spectroscopy, recorded on the ^13^C nucleus. Spectra of DNA in the absence and presence of Hfq-CTR. (**A**) Solution NMR spectrum of double-stranded (dA:dT)_59_. (**B**,**C**) Solid-state NMR insensitive nuclei enhanced by polarization transfer (INEPT) spectra of ds (dA:dT)_59_/HFq-CTR complexes with a 2:1 and 1:1 DNA:Hfq-CTR molar ratio, respectively. All spectra were referenced according to the DSS signals.

Solution and solid-state NMR were then used to monitor the (dA:dT)_59_ DNA behavior during complex assembly. Figure 4A shows the spectral ^13^C fingerprint of double-stranded (dA:dT)_59_ DNA in solution, reflecting the signal distribution of all DNA carbon atoms. Solid-state NMR INEPT spectra, recorded on the ^13^C nucleus, report on the mobile carbon moieties in a sample, including ^13^C-containing molecules in solution and flexible moieties in a molecular assembly (Figure 4B,C). When the dsDNA is present during the assembly of Hfq-CTR filaments, its propensity to associate to the filaments or to remain in solution should be determined by its affinity to the filaments, as well as by the steric effects experienced upon association. The ssNMR spectra displayed in Figure 4B,C monitor the presence of mobile (dA:dT)_59_ dsDNA and Hfq-CTR moieties when dsDNA is present at a DNA/Hfq-CTR mol/mol ratio of 2:1 (B) and 1:1 (C) during the Hfq-CTR assembly. We observe a significant signal of Hfq-CTR indicative of soluble monomers or comparatively small multimeric states, deposited during filament centrifugation that represent the filament-monomer equilibrium. The signal intensity of the soluble peptides between the two conditions, i.e., complex assembly in a 2:1 or 1:1 DNA:Hfq-CTR ratio, is comparable, indicating that the amount of Hfq-CTR peptides in solution is equal. We can therefore assume that doubling the amount of DNA has not shifted the equilibrium of monomeric Hfq-CTR versus Hfq-CTR in filaments. When the dsDNA is prepared in a 2:1 molar ratio with Hfq-CTR during filament formation, we can assign all DNA signals that are not obscured by the peptide signals (B). The signals reveal the presence of isolated, soluble dsDNA. In contrast, when using a molar ratio of 1:1 dsDNA/Hfq-CTR, the signals of the (dA:dT)_59_ DNA are absent (C). The absence of DNA signals in the ssNMR spectrum indicates that all DNA strands are insoluble to the limit of detection and involved in higher order complexes, reflecting their association to peptide filaments.

## 4. Discussion

In addition to its role in riboregulation, Hfq binds to DNA and plays a relevant role in bacterial chromatin organization. As Hfq has been reported to have a substantial impact on transcription and replication processes, a characterization of the complex physico-chemical and structural properties of Hfq:DNA association and organization is necessary. In this manuscript, we evidence a new property for Hfq as a DNA shaper; Hfq can nucleate bacterial chromatin alignment, similarly to the H5 histone in eukaryotes [54]. Hfq was previously shown to interact preferentially with specific (A-rich) DNA sequences [13,14] and it was proposed that the protein first nucleates these sequences to propagate to surrounding regions, similarly to H-NS NAP [55]. Consequently, Hfq potentially covers up local regions of DNA, due to cooperative fiber-like pattern generation in vitro and presumably also in vivo [14,15,17]. Taking into account that the *E. coli* nucleoid contains 10–15 µM of Hfq on average, significantly higher localized concentrations are found [12,17]. These high local concentrations would provide good potential for Hfq binding DNA.

Using SRCD and SRLD, we demonstrated that Hfq affects the DNA base tilt, i.e., the angle of the planar bases with respect to the helical axis within the double helix. The base tilt of free (dA:dT)_59_, which is similar to the poly[d(A)]-poly[d(T)] long polymer, may be inclined differently by a few degrees when bound to the protein, similar to the base tilt observed for free poly[d(AT)]-poly[d(AT)] [49]. This change in base tilt may result in a change of the distances between consecutive phosphates and results in a ribbon-like structure that impacts DNA bending. Previous works showed that Hfq changes DNA persistence length and bending [29]. Here we furthermore show that this change in bending influences DNA rigidity and local alignment. We assume this effect is specific to Hfq-CTR and not general for amyloids, as α-Synuclein binding to DNA does not result in such an alignment [56]. Nevertheless, the relevant amino acids of Hfq-CTR involved in DNA binding remain to be identified. According to the type of interaction involved in the formation of the complex (hydrogen-bonding, see [29]), we suspect an important role of the abundant histidine and serine residues and plan to further analyze corresponding mutants to observe functional consequences on the formation and nature of the complex.

**Figure 5:**
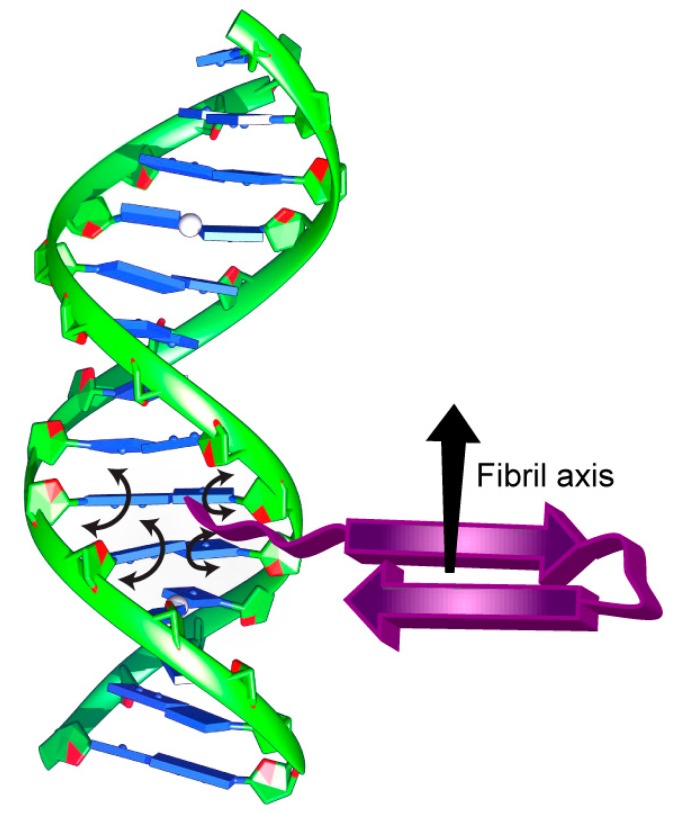
Schematic representation of DNA bound to the Hfq-CTR amyloid region highlighting the suggested impact of Hfq-CTR on the base tilt with arrowheads.

In parallel, our solid-state NMR results confirm the SRLD and SRCD spectroscopy results. During the formation of filamentous Hfq-CTR/DNA complexes, the peptide is saturated in a 1:1 DNA:Hfq-CTR ratio because the ssNMR signal does not decrease. The absence of DNA signals in the ssNMR INEPT spectra, when prepared in 1:1 complexes, shows that all DNA is involved in the structural elements of the filament core. However, once the peptide involved in DNA/peptide complexes is saturated, no additional complexes can be formed, as reflected by the constant peptide signal and the appearance of DNA moieties in the spectra. Assuming that at an approximate 1:1 DNA:Hfq-CTR ratio all DNA strands participate in Hfq-CTR/DNA complex formation, the orientation of the peptide filament axis with respect to the DNA strand is sterically restrained. Considering the area that would be occupied by (dA:dT)_59_ dsDNA along the strand axis versus in an orthogonal arrangement, the approximate 1:1 dsDNA:Hfq-CTR association suggests an out-of-axis organization of the two elongated molecular architectures, permitting an optimal packing. This is indeed confirmed by SRLD measurements on the dsDNA:Hfq-CTR complex, where the β-strand is found to be perpendicular to the dsDNA helical axis. This would, for example, be the case if the peptide arranges in the DNA grooves or associates in an orthogonal orientation to the dsDNA, assuming an amyloid arrangement (Figure 5).

Functionally, such a property could generate short-range chromosomal contacts and could be of major importance for some DNA-related processes such as homologous recombination, site-specific recombination or DNA repair, with an impact on evolutionary processes and adaptation to environment [57,58,59]. Homologous recombination for instance could be one of the main evolutionary mechanisms of bacterial genomes [60]. This property may also have a role in plasmid replication and in the exchange of genetic information [15,20]. Moreover, several proteins involved in promoting amyloid disorders, such Alzheimer’s, Parkinson’s, and Creutzfeldt-Jakob diseases, have DNA-binding properties [61]. Considering the amyloid-like character of Hfq, our work suggests that DNA alignment by an amyloidogenic protein may be a way to alter the expression profiles of disease-related genes [62]. Finally, the property of shaping DNA with amyloid-like protein filaments, providing stiffness at the micrometer-scale, could be used as an asset to extend nucleic acids-based origami formations at larger scales [63].

## 5. Conclusions

Although a lot of work remains to be done to decipher the various Hfq mechanisms at stake *in vivo* and the structural and mechanistic background *in vitro*, our results definitively evidence that Hfq and precisely its CTR has an important role to be considered as bacterial chromatin organizer and in DNA-related regulative processes. Different reports based on *hfq* gene-disrupted cells revealed the important regulatory role of Hfq in various cellular processes, with major impact on environmental adaptation. Most of these effects were attributed to sRNA-Hfq-based regulation [15,18,64], but additional direct effects on DNA-related processes of Hfq should definitely be considered in the future.

## Supplementary Materials

Supplementary materials can be found at https://www.mdpi.com/2076-2607/7/12/639/s1. Figure S1: AFM analysis of Hfq-CTR fibrils in the absence of DNA.

## Abbreviations

AFM	Atomic force microscopy
CTR/NTR	Hfq C/N-terminal region
INEPT	insensitive nuclei enhanced by polarization transfer
nt	nucleotide
sRNA	small regulatory noncoding RNA
ssNMR	solid state NMR
SRCD/SRLD	Synchrotron Radiation Circular/Linear Dichroism
