# Supplementary material for: The Bacterial Amyloid-Like Hfq Promotes In Vitro DNA Alignment
Source: Microorganisms. 2019 Dec 3;7(12):639. doi: 10.3390/microorganisms7120639 (PMC6956100; doi:10.3390/microorganisms7120639)
Supplement: Supplementary file 1 [file microorganisms-07-00639-s001.zip › SupFigS1Wienetal.pptx]

## Slide 1
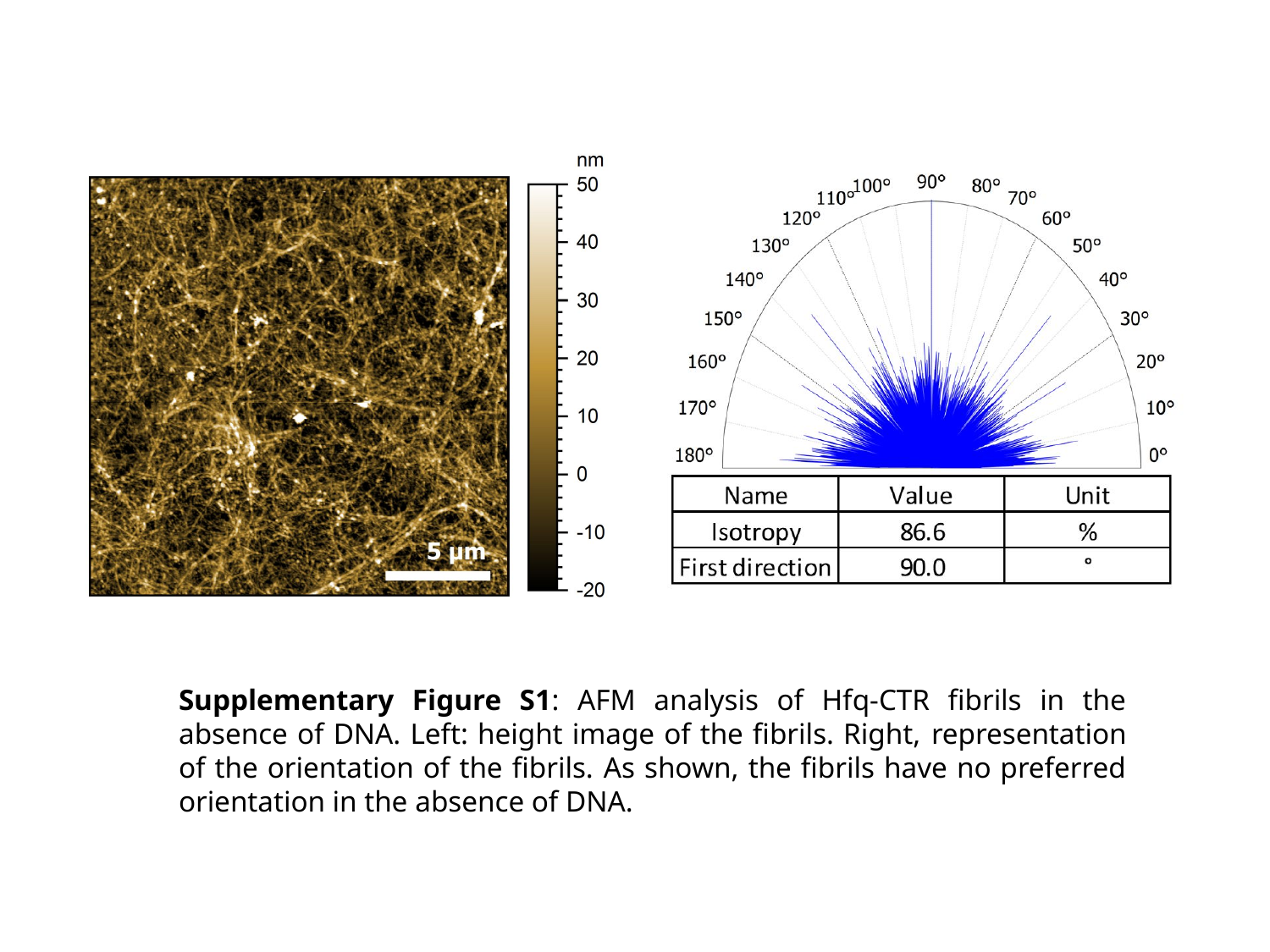

Supplementary Figure S1: AFM analysis of Hfq-CTR fibrils in the absence of DNA. Left: height image of the fibrils. Right, representation of the orientation of the fibrils. As shown, the fibrils have no preferred orientation in the absence of DNA.
